# Supplementary material for: Are carbon emissions trading and green financial instruments synergistic? -Comprehensive quantitative research based on content analysis
Source: PLoS One. 2024 Mar 7;19(3):e0298601. doi: 10.1371/journal.pone.0298601 (PMC10919601; doi:10.1371/journal.pone.0298601)
Supplement: S1 Data — (ZIP) [file pone.0298601.s001.zip › Data/地级市Z_F工作报告.zip!/┴╔─■/╕o╦│╩╨/└┤╫╘╣1⁄2╓┌║┼í╛┬φ┐╦╩2╛▌═°í┐_2012.docx]

各位代表：

　　现在，我代表市政府向大会报告工作，请予审议，并请市政协各位委员提出意见和建议。

一、2011年政府工作回顾

　　刚刚过去的一年，面对极为复杂的国内外形势，在省委、省政府和市委的正确领导下，在市人大和市政协的监督支持下，市政府团结带领全市人民，深入贯彻落实科学发展观，做大经济总量，做优生态环境，做实民生工程，完成了市十四届人大四次会议确定的目标任务，实现了“十二五”的良好开局。

　　预计地区生产总值完成1127亿元，同比增长16%；地方财政一般预算收入106.5亿元，增长31%；全社会固定资产投资800亿元，增长32%；社会消费品零售总额390亿元，增长17%；城市居民人均可支配收入18050元、农民人均纯收入8500元，分别增长18%，实现了“两个高于”的目标。特别是地区生产总值突破千亿元，地方财政一般预算收入突破百亿元，经济和社会发展综合指数由上年全省的第8位跃至第4位，我们可以自豪地说，抚顺人期盼已久的晋位赶超、重铸辉煌的那一天，已经指日可待。

（一）区域发展呈现新局面

　　一年来，我们深入实施“两城两带”和县区跨越发展战略，全市多点支撑的格局基本形成。

　　沈抚同城化加速推进，抚顺沈抚新城继续走在沈阳经济区38座新城、新市镇前列，地区生产总值完成230亿元、财政一般预算收入突破23亿元，均接近全市总量的四分之一。石化新城产业提升、城市更新和基础设施建设均开历史先河。南环产业带一批重大项目开工建设。以“五点一线”为重点的浑河景观带建设取得重大进展，抚顺的白天生机盎然，夜晚流光溢彩。

　　县区大力实施三年倍增计划，清原县首次跻身财政一般预算收入10亿元县的行列；顺城区、新抚区财政一般预算收入均超过13亿元。县区经济发展呈现勃勃生机。

（二）产业结构明显优化

　　一年来，我们全力转变发展方式、调整产业结构，经济发展的质量和后劲显著提升。

　　工业支撑作用增强。预计完成规模以上工业增加值580亿元、增长16%，对全市经济增长贡献率达到52%。亿元以上工业项目新开工158个、竣工97个，均增长40%以上。其中，抚顺石化千万吨炼油工程、抚矿百万吨造纸第一条生产线、抚顺铝业30万吨电解铝改造竣工投产。沈抚新城工程机械及制品、石化新城化工新材料两大产业集群，实现产值820亿元、增长56.5%。抚挖重工“千吨级超大型履带起重机”项目列入国家“863”计划，罕王控股公司成功上市，民营经济占全市经济比重超过60%。

　　服务业发展步伐加快。预计完成服务业增加值370亿元、增长16%。积极打造沈阳经济区购物天堂和抚顺中央商务区，旺力商贸城、万达广场等现代商贸和城市综合体项目顺利推进，大商抚顺集团、浙商国际商贸城向多业态大型流通企业集团迈进。房地产业重在品质提升，万科、绿地、恒大等国内行业龙头企业进入抚顺，全年完成房地产开发投资112.4亿元、增长32.5%，商品房销售额126.1亿元、增长62.7%。旅游业品牌效应凸显，红河大峡谷漂流、皇家海洋世界等闻名遐迩，丰远?热高乐园巴厘岛水世界建成营业，2011中国（抚顺）满族风情国际旅游节成功举办，抚顺旅游知名度和吸引力迅速提升，旅游总收入增长20%。

　　特色农业快速发展。大力发展特色主导产业，特色产业产值占农业产值比重达到45%。抚顺县新建以单片黑木耳为主的食用菌产业基地15个，新增食用菌4000万袋、总量达到7000万袋；清原县新增中药材24万亩、总量达到86万亩，新增百合花种植大棚310栋、户均增收2.5万元；新宾县新增香菇3000万袋、总量达到1.7亿袋。畜禽标准化规模养殖比重达到62%、比上年提高9个百分点。坚持特色农业与旅游相结合，大力发展农家乐，成功举办第五届水果采摘月，接待游客和销售收入均创历史新高。

　　（三）改革开放成效显著

　　一年来，我们强力推进国企改革和对外开放，盘活存量、引进增量，培育了一批新的经济增长点。

　　全面完成以抚顺铝厂、抚钢集团为重点的155户国企改制攻坚任务，基本解决以企业职工“一金两险”为重点的138户已改制企业遗留问题。国企改革历经10年，终于画上了句号，了结了一段历史。

　　突出主题概念招商，“两城两带”、“五点一线”成为客商关注的热点，德国汉圣化工集团等行业龙头企业进驻抚顺。全年引进域外资金480亿元，增长40%；开工建设亿元以上重点项目394个，完成投资584亿元、增长22.5%。

　　向上争取支持，累计争取资金75亿元，争取土地指标1243公顷、增长118%。争取国家资源枯竭城市转型支持政策延长5年，可获得支持资金15亿元；在市人大代表的积极努力下，争取全国首家矿山地质灾害综合治理试点市工作取得重大进展。

　　（四）城市环境日新月异

　　一年来，我们坚持城市建设与管理并重，大手笔还绿、还畅、还净、还美于城，城市面貌焕然一新。

　　加大城市建设力度。城建投入首次突破100亿元。拆迁旧城区95.7万平方米，增长2.5倍。城市绿化覆盖率达到40.8%、比上年提高1.3个百分点，人均公共绿地面积新增1.2平方米、达到9.2平方米，荣获“省级园林城市”称号。完成月牙岛生态公园一期、沈抚新城金风湾等十大公园建设改造。大修装饰7座桥梁，改造34条城区道路，启运大道、东环大道竣工通车。新建改造4座污水处理厂，关停45家重污染企业，拆除46台燃煤小锅炉，全面完成节能减排指标。电网建设投资近13亿元，创造历史最好水平。

　　加大城市管理力度。市本级新增环卫设备投入1000万元，购置大型环卫车辆23台；增加环卫工人552人，全市大街小巷的清洁度迈上新台阶。综合整治市容环境，拆除违章建筑8835处，规范占道市场24个，车辆泄漏遗撒和脏乱差问题得到有效治理，顺利通过省级卫生城复核验收。“数字抚顺”建设通过国家验收，抚顺被授予“全国数字城市建设示范市”和国家“天地图市级节点建设示范市”。

　　（五）民生工程全面推进

　　一年来，我们全方位保障和改善民生，幸福抚顺建设取得初步成效，多项工作走在了全省前列。

　　健全社会保障体系。在全省率先实现新型农村养老保险全覆盖，稳步推进城镇居民养老保险；强力推进社保扩面征缴，养老保险统筹基金征缴21.85亿元、增长30%，确保了全市31万名企业离退休人员、53亿元基本养老金按时足额社会化发放；三级以上医院城镇职工基本医疗保险住院人均定额结算标准，平均提高1000元；提高了城镇居民基本医疗保险、新农合参保的政府补助标准，平均每人每年由120元增至200元；将3.3万名国有、集体企业工伤人员，全部纳入工伤保险统筹管理。坚持以创业带动就业，全市城镇实名制就业9.6万人，登记失业率控制在4%以内。

　　强化社会救助。继续推进80个贫困村整村扶贫、7.78万人脱贫，扶贫攻坚两年任务一年完成；继续提高低保救助标准，城市低保和月人均救助标准均高于全省平均水平，城乡低保对象领取临时物价补贴4000多万元；继续加大扶贫救助力度，提高孤儿养育标准，医疗救助245名患重大疾病的贫困妇女儿童，资助1832名贫困学生，救助2000户贫困村民，建设100套农村单亲贫困母亲安居住房，免费为2万多名贫困妇女进行健康检查；社区工作者每人每月生活补贴提高200元。

　　改善居住环境。全面完成了市区现有的192条“摸黑路”改造任务。投入4500万元，解决了棚改小区自来水管线爆裂、屋面漏雨等历史遗留问题。大力推进棚户区改造、廉租房和公租房建设，惠及1.1万户、3.5万人，保障性安居工程建设走在全省前列。

　　应对抚电公司因地质灾害紧急关停，我们果断决策、周密部署、迅速行动，仅用2个月的时间，就完成了应急替代热源建设，确保了城区600万平方米、10万户、30万人的冬季供暖，创造了抚顺工程建设史上的一大奇迹。

　　（六）社会事业协调发展

　　一年来，我们大力发展社会事业，努力满足人民群众日益增长的文化生活需求。全年投入资金9.6亿元、增长28.3%。

　　坚持优先发展教育，新增1.56亿元改造提升42所城区薄弱小学和幼儿园，新建和改扩建13所农村乡（镇）中心幼儿园，基本完成中小学校舍安全工程，抚顺县、望花区、沈抚新城“双高普九”通过省级评估验收，四方高中成为省示范高中，一职专成为国家级中等职业教育改革发展示范学校。加强校长和骨干教师的交流培训，教育教学质量进一步提高。

　　丰富群众文化生活，成功举办“红诗红歌?唱响抚顺”、首届群众文化艺术节、第四届读书节和“迎国庆、爱家乡”月牙岛游园周等主题活动，群众性文化娱乐活动丰富多彩。

　　加快文化设施建设，沈抚新城规划展示馆、抚矿集团工业博物馆竣工，抚顺文化市场正式运营，抚顺剧院等重点项目进展顺利，沈抚新城文化产业园被列为“省级文化产业园区”。

　　促进卫生事业发展，基本药物制度改革稳步推进，市中医院、第二医院的建设改造工程顺利实施，新宾县中心医院和6家社区卫生规范化服务中心建成投入使用。

　　成功举办了抚顺市第六届运动会、第三届全民健身节，承办了全国少年乒乓球锦标赛、辽宁省第八届三块石登山比赛，群众体育活动蓬勃发展。

　　社区条件进一步改善，新增办公用房近7000平方米，超过半数的社区办公用房达到400平方米以上。“平安抚顺”建设扎实推进，信访总量明显下降，安全生产形势总体稳定。

　　全市民族团结，宗教和谐。军民融合式发展稳步推进，民兵预备役工作进一步加强。其它各项工作也都取得了新的进展。

　　（七）政府建设不断加强

　　一年来，我们围绕加快发展和改善民生两大主题，讲大局、讲责任、讲效率、鼓干劲，在建设人民满意政府上又前进了一步。

　　自觉接受市人大及其常委会的法律监督、工作监督和市政协的民主监督，办理342件人大代表建议、447件政协提案，办复率100%，解决率58.7%；畅通社情民意渠道，受理5.2万件次市民电话诉求，回复率100%，解决率97%。

　　强化正面激励和绩效考核，广大公务人员的工作激情、工作干劲和工作效率进一步提升。全市上下政通人和、心齐气顺、干事创业的氛围业已形成；各县区、各部门、各单位团结进取、比学赶超、竞相发展的态势业已形成；抚顺人关心家乡、热爱家乡、甘为家乡作贡献的风气业已形成。

　　加强标本兼治和综合治理，特别是对土地出让、工程招投标等重点领域和关键环节，实施严格的管理、监督和检查。民心网、政风行风热线受理3000余件群众反映问题，办结率和群众满意率均超过96%。

　　各位代表：一年来的实践表明，办好抚顺的事情必须牢牢把握以下几点：一是一心一意谋发展。我们时刻牢记“解决所有问题的关键是发展”，坚持一切从抚顺实际出发，一切服从服务于发展，切实增强预见性、强化系统性、提高针对性，不断解放思想、完善思路举措、健全工作机制、创新工作方式，促进地区加快发展。二是尽心竭力惠民生。我们始终坚持群众利益高于一切，全方位改善民生，并在投入规模、涵盖领域、受益人数上均创历史新高。三是真抓实干重效率。我们本着对抚顺历史和人民高度负责的态度，坚持科学决策和高效决策、制定战略与细化战术、明确责任同监督考核相结合，做到工作部署高标准、任务落实高效率，说了就算，定了就干，干就干好。四是激发热情鼓干劲。我们坚持责权利挂钩，强化目标管理和奖优治庸，支持鼓励各级干部充分发挥聪明才智，创造性地开展工作，涌现出了一大批想干事、能干事、干成事的干部。

　　成绩固然可喜，但发展中的问题更须理性应对。一是困难群体大，贫困人口多，一些群众的生活还很艰难。二是城市空气污染、水污染的问题还比较严重，教育、就业、住房、社保等与百姓的期待还有一定距离。三是工业产业集群规模小、产业链短，产业丰厚度和完整度亟待提高；服务业占地区生产总值比重较低，产业规模和档次与百万人口城市还不相称。四是县区经济总量偏小，发展不够充分，与省内先进地区相比还有较大差距。对此，我们将采取有力措施，认真加以解决。

　　回首过去的一年，抚顺的变化有目共睹，抚顺的发展令人鼓舞。这些成绩的取得，是省委、省政府高度重视、亲切关怀的结果，是市委科学决策、正确领导的结果，是市人大、市政协有效监督、大力支持的结果，是全市230万人民艰苦创业、团结奋斗的结果。在此，我代表市政府，向市人大代表、政协委员，向各民主党派、工商联、无党派人士和人民团体、离退休老同志，向驻抚人民解放军、武警部队官兵、公安干警和中省直单位，向所有关心抚顺发展的国内外朋友，向全市各族人民，表示崇高的敬意和衷心的感谢！

二、2012年政府工作目标与任务

　　2012年是实施“十二五”规划承上启下的重要一年，也是我市巩固发展态势、赢得更大发展的关键一年。我们必须清醒地认识到，当前国际国内经济形势十分严峻复杂，国际市场持续低迷，国内经济下行压力加大。今年特别是上半年，发展要素的制约将更加严重，经济发展将会十分困难。同时更应该看到，我们对经济变化认识较早，行动较快，准备较充分，争取了工作的主动权。最为关键的是，我市政通人和，凝心聚力、大干快上的氛围业已形成。只要我们切实增强忧患意识和风险意识，切实增强“发展是解决抚顺所有问题根本途径”的理念，善于运用有利条件和积极因素，抓准抓好抓住战略机遇期，就完全可以把我们自己的事情做得更好，完成全年“两个高于”的奋斗目标，实现抚顺经济的强劲发展和在全省位次的弯道超越。

　　全市经济社会发展的总体思路是：以邓小平理论和“三个代表”重要思想为指导，深入贯彻落实科学发展观，深刻把握“稳中求进、进中求好、好中求快”的总基调，深入实施“两城两带”和县区跨越发展战略，大力发展工业、服务业和县区经济，着力推进生态城市建设，全力促进民生改善和社会和谐，为建设幸福抚顺奠定坚实基础。

　　全市经济社会发展的主要预期目标是：地区生产总值增长16%，地方财政一般预算收入增长25%，全社会固定资产投资增长32％，社会消费品零售总额增长17%，城市居民人均可支配收入增长16%，农民人均纯收入增长15%，万元生产总值能耗降低3.9%。

　　为实现上述目标，重点抓好九个方面工作。

　　（一）继续举全市之力建设沈抚新城和石化新城

　　沈抚新城、石化新城是抚顺应对挑战、强劲发展的重要支撑，今年要确保发展速度更快、建设水平更高，成为全市经济发展的重要增长极。

　　沈抚新城要突出“新”字，围绕建设国际知名、国内一流的现代高端城市，加快“三区”建设发展。产业区，要依托国家先进能源装备产业化基地，全力打造工程机械及制品、钢铁材料深加工产业集群，尽快壮大新兴工业产业。核心区，要依托毗邻沈阳的区位优势，全力打造沈阳经济区购物天堂，尽快形成新的消费热点区域。生态区，要依托皇家海洋世界和丰远?热高乐园等重点旅游项目，全力打造沈阳经济区休闲娱乐天堂，尽快形成新的旅游产业集聚区。全年沈抚新城生产总值增长41%，财政一般预算收入增长40%，全社会固定资产投资增长45%。

　　石化新城要突出“变”字，围绕建设国家级化工新材料基地和现代化新城区，在产业发展、城市更新和民生改善上迈出更大步伐。工业区，要全面完成高新区4.2平方公里基础设施及公用设施建设，加快推进总投资120亿元的69个重点项目建设，化工新材料产业集群初具规模。核心区，要加快推进“4桥7路1坝1园”等重点工程，全面完成28平方公里基础设施建设，“四纵六横”交通网络基本形成。生态区，要充分利用自然生态和历史文化的资源优势，全力推进“温泉水世界”等重点项目建设，着力扩大净月湖及萨尔浒风景区的对外影响力。全年石化新城生产总值增长51%，财政一般预算收入增长28%，全社会固定资产投资增长50%。

　　（二）着力壮大工业经济

　　坚持工业强市不动摇，以增量带动产业优化，以科技促进产业升级，全面提高工业总量和质量，规模以上工业增加值增长18%。

　　抓好工业经济运行。加强预警分析，对20户重点工业企业进行动态监控，促其有序组织生产。加强要素保障，强化煤电油运等生产要素的协调调度，确保重点行业和重点企业开足马力生产。鼓励兼并重组，培育一批销售收入超100亿元的骨干企业。

　　做大工业产业集群。按照有特定空间、有主导产品、有规模、有品牌、有产业链、有各种服务平台的内涵要求，加快推进12个重点产业集群建设。采取以奖代补、贷款贴息等方式，加大产业园区基础设施建设的投入，尽快实现道路和场地“一通一平”，满足项目落地的基本条件。全年工业产业集群实现产值1540亿元，增长40%。

　　做强传统优势产业。石化产业，积极推进石油一厂、化塑厂等企业的搬迁改造，加快百万吨乙烯项目竣工投产，打造世界级炼化基地。煤炭产业，加快推进50万吨页岩油化工、2×30万装机热电和百万吨造纸项目建设，推进产业结构调整。冶金产业，全面启动新钢铁搬迁改造和产品结构调整，积极支持抚特钢百亿技改项目，实现铝资源深加工的突破。装备制造产业，大力支持隆基磁电、高科电瓷等重点企业的改造和发展，进一步增强核心竞争力。

　　做精中小微企业。继续实施“启明星”计划，大力支持成长型中小微企业技术改造和科技创新，激活企业发展的内在动力。强化县区政府是发展中小微企业的责任主体，完善贷款担保体系，引导支持全民创业，培育更多的本地“小老板”。加强组织和培训，帮助中小微企业开发市场，特别是为本地大企业搞好配套服务。中小微企业实现工业总产值1600亿元，占全市工业总产值的60%。

　　做优科技支撑平台。充分发挥抚顺石油化工研究院、煤科总院沈阳研究院、辽宁石油化工大学的优势，重点依托沈抚新城和石化新城两大特色产业基地，加强产学研合作，搭建企业公共技术服务和科技成果转化平台，增强科技对经济的支撑力。全年高新技术产业增加值增长40%以上，国家高新技术企业数量增长50%以上。

（三）加快发展服务业

　　服务业是优化经济结构、促进消费、增加就业、惠及民生的重要产业，我们有条件实现发展的新突破。全年服务业增加值增长15%。

　　大力发展商贸流通和现代服务业。一是加快服务业集聚区建设，每个城区至少形成一个年销售额20亿元的商贸集聚区。二是加快市场流通体系建设，重点推进榆林采煤沉陷区专业市场群建设，支持大型商贸企业连锁经营。完善新农村流通服务网络，实施农超对接，降低农产品的流通成本。三是加快现代服务业发展，完善多元化投融资服务体系，鼓励商业银行增加信贷，支持抚顺银行做大做强，推动现代物流业发展。

　　稳步发展房地产业。针对抚顺房地产居民刚性需求大、发展态势健康的实际，坚持住宅地产、工业地产、商业地产同步推进，坚持旅游地产、教育地产、文化地产全面发展，加大城市更新力度。全年旧城区拆迁改造100万平方米，房地产开发投资额和商品房销售额均增长15%以上。

　　发展壮大旅游业。围绕打造旅游目的地城市，强化旅游与文化、教育、体育、特色农业等有机结合，推进吃、住、行、游、娱、购全面发展。依托5个国家级森林公园，做好“山”的文章；依托浑河水系等丰富的水资源，做好“水”的文章；依托清前史迹、满族民俗、雷锋精神和工业文明等特色资源，做好“文化”的文章，推出山、水、文化旅游综合体和旅游线路。完善旅游基础设施，推进丰远城堡、君悦帝豪等五星级酒店和商务酒店建设。加大宣传推介力度，不断提升抚顺旅游的知名度和吸引力。全年旅游总收入增长20%以上。

　　（四）全面推进县区经济大发展

加快县域经济发展。三县地区生产总值增长25%，地方财政一般预算收入增长35%，固定资产投资增长40%。一是推进工业产业集群建设。清原县要加快推进特种输变电设备和再生资源产业集群建设，实现产值50亿元。新宾县要加快推进钙化物制品、焊丝新材料和南杂木特种材料产业集群建设，实现产值30亿元。二是推进特色农业发展。壮大“一县一业”，加快发展林下经济及中药材、食用菌、百合花等主导特色农业，积极推进现代畜牧业示范区和6个超亿元的产业化龙头企业建设，特色农业产值占农业产值比重达到50%以上。深化集体林权配套改革，规范和鼓励森林资源流转，促进农民增收。三是推进县城和重点小城镇建设。全面启动抚顺县县城建设，争取成为国家新型城镇化试点示范项目；提升清原县、新宾县县城的规划建设水平，拓展发展空间，完善功能布局，房地产新开工面积均达到40万平方米。大力推进永陵、南杂木、红透山、后安等重点小城镇建设，增强辐射、带动和承载能力。四是推进县域旅游业发展。重点打造清原县生态旅游、新宾县兴京文化旅游和抚顺县休闲农业旅游产业集聚区，三县旅游总收入增长22%以上。五是推进新农村建设。加大农村基础设施建设力度，抓好农村新能源建设和环境综合整治，改善农民生活质量，加速推进城乡一体化发展。

　　加快城区经济发展。一是打造商贸服务业集聚区。重点推进新抚区中央商务区、望花区丹东路中部商贸集聚区和顺城区新华商贸集聚区建设，实现主营业务收入161亿元、增长24%。二是建设工业产业集群。新抚区要加快建设页岩油化工产业集群，实现产值10亿元。望花区要加快建设包装材料和有色制品产业集群，实现产值20亿元。顺城区要加快建设起重装备产业集群，实现产值30亿元。三是发展城区旅游。围绕建设都市旅游产业集聚区，新抚区重点依托天鹅湖发展文化休闲游，望花区依托雷锋纪念馆发展红色文化游，顺城区依托高尔山公园发展宗教文化和生态休闲游。全年城区旅游总收入增长18%以上。

　　（五）强力扩大对外开放

　　牢固树立“越困难越要抓开放”的理念，通过抓开放、抓项目来激发活力、增强动力、壮大实力。

　　强化招商引资。围绕“两城两带”、特色资源深加工、打造产业集聚区，策划、包装和推出一批主题招商概念，打出招商旗帜。强化“一把手”招商和招商队伍建设，坚持走出去与请进来相结合，努力吸引知名企业投资抚顺，实现对外开放新突破。全年引进亿元以上项目130个，直接利用外资5.5亿美元，域外引资到位额625亿元、增长30%。

　　向上争取支持。用好用足国家和省的财政、金融、土地、产业政策，力争抚顺成为全国首家矿山地质灾害综合治理试点市。

　　破解发展瓶颈。坚持集约节约用地，用好采矿用地改革试点、城乡建设用地增减挂钩等政策，力保重点项目建设用地。扩大直接融资，鼓励企业上市。增加间接融资，全年新增贷款160亿元、增长1倍，新增小额贷款公司、融资性担保公司各5户，鼓励发展新型农村金融机构。

　　抓实项目建设。强化服务支持，促进签约项目尽快落地、在建项目早日达效，以抓项目推动大发展。全年开工建设超亿元重点项目443个，完成投资700亿元。

（六）加快建设生态宜居城市

　　围绕建设“国家森林城市”和“生态城市”，继续加大投入，着力实施“六大工程”，让抚顺青山常在、碧水常流、蓝天永驻，让抚顺环境清新、交通顺畅、街路整洁，让百姓的生活更舒心、更惬意、更幸福。

　　实施青山工程。加大退耕还林力度，全面清理小开荒，加快关闭矿山的生态恢复治理，突出整治公路沿线和城市周边损坏山体，着力打击破坏森林资源等违法行为。开展大规模植树造林，提高浑河两岸、主干街路和旅游线路等重点区域的绿化水平，全年完成绿化207万平方米，绿化总量比上年翻一番。

　　实施绿水工程。加快污水收集管网等基础设施建设，完成三宝屯污水处理厂扩建和海城污水处理厂建设，启动污水处理厂污泥处置及演武垃圾厂渗滤液处理工程，新建城乡污水处理厂12座、乡镇垃圾处理场7座，农村环境连片整治158个行政村。重点整治浑河城市段和13条支流河，建设百里生态走廊，打造水清景美的景观河。

　　实施蓝天工程。加快集中热源建设，拆除50台燃煤小锅炉，扩大城区集中并网供热覆盖面；强化污染物减排，推进城区“退二进三”，综合整治大气污染严重的地区和企业，着手解决群众反映强烈的顺城区龚家沟环境污染和消防安全隐患等问题。

　　实施景观工程。按照恢复生态环境、保留工业遗址的原则，编制抚电公司、石油一厂等搬迁企业腾空地的规划。完成月牙岛生态公园、高尔山公园等重点景观续建工程，建成石化新城好运角湿地公园和沈抚新城玉露潭水系景观走廊。

　　实施畅通工程。完成抚通、桓永高速公路建设，续建萨尔浒大道，建设普通公路510公里，改扩建永济路、202线等重点道路，大规模改造城乡结合部、小街小巷道路。甲邦大桥、东洲河口桥和章党大桥建成通车。新建沈抚新城综合枢纽站。积极推进投资15.8亿元的电网项目建设，实施15公里供水输水渠道改造工程，完成42.5公里天然气管网建设。

　　实施清洁工程。深化环境卫生精细化管理，强化市政基础设施维修、养护和管理，综合整治12条重点街路景观环境，创建5条城市管理示范街路。加强居民小区物业管理。深入开展城区清洁行动，街路人工清扫保洁率、生活垃圾清运率均达到100%。

　　（七）大力建设文化强市

　　充分发挥抚顺特色文化优势，以满足人民群众精神文化需求为出发点和落脚点，坚持文化发展与经济发展并重，把文化建设纳入经济社会发展总体规划，统一实施、共同推进，推动抚顺文化事业繁荣发展。

　　加强文化基础建设。建成抚顺大剧院、市民文化活动中心、沈抚新城文化中心，启动前清博物馆建设，推进县区文化广场、图书馆、文化馆和社区文化书屋建设，扶持沈抚新城特色文化产业基地、新宾上夹河特艺文化产业基地、新抚天鹅湖文化产业和千金乡影视基地建设，不断提升文化设施的承载能力，尽快将抚顺的文化优势转化为经济优势。

　　丰富群众文化生活。深入挖掘富有抚顺特色的文化资源，宣传弘扬“创业、奉献、和谐、争先”的抚顺精神，围绕创建“雷锋城”，创作生产更多、更好的精神文化产品，打造独具抚顺特色的群众文化品牌。广泛开展群众性文化活动，举办雷锋歌曲群众歌咏大会、首届冰雪节、戏水节、月牙岛中秋之夜专场演出、迎接“十八大”综合歌舞晚会等大型主题文化活动，让抚顺人民享有更加健康丰富的文化生活。

　　（八）全力构建和谐社会

　　更加注重民生改善。一是坚持把稳定就业作为最根本的民生，大力扶持自主创业和自谋职业，确保城镇实名制就业9.5万人、零就业家庭动态为零。二是坚持把社会保障作为最普遍的民生，继续强化社保扩面征缴，确保33.9万名企业离退休人员、65亿元基本养老金按时足额社会化发放，扩大城镇职工和城镇居民养老保险覆盖面，力争城镇职工、城镇居民和困难企业退休人员医疗保险全覆盖；进一步提高城乡低保标准、农村五保供养标准和重点优抚对象生活补助标准；提高新型农村合作医疗报销比例和最高支付限额。三是坚持把扶贫解困作为最紧迫的民生，继续推进扶贫攻坚，大力发展慈善事业，强化对贫困农民、低保家庭精神障碍患者、低保家庭大学生和患重大疾病贫困妇女儿童的帮扶救助。四是坚持把改善群众生活条件作为最基本的民生，继续加强保障性安居工程、农村饮水工程建设和老城区供水设施改造；有序推进“菜园子”建设；维护市场秩序，保持生产生活必需品市场价格的基本稳定；加快建设改造社区、村屯的街路和广场，扩大绿化、硬化的覆盖范围；完成市殡仪馆的搬迁改造。

　　更加注重社会事业。一是坚持教育优先，重视发展学前教育；扎实推进义务教育均衡发展，投资2亿元提升改造初级中学，全面完成“双高普九”达标验收；强化普通高中优质特色建设和职业教育优化发展；支持高等教育加快发展，重点推进抚顺职业技术学院和师专整合搬迁，新校舍主体建设年内竣工。二是深化医药卫生体制改革，加快公共卫生服务体系建设，健全覆盖城乡的基本医疗制度，推进市中医院、第二医院的建设改造，不断改善群众就医条件。做好人口和计划生育工作，开展“幸福家庭”的创建活动。三是实施体育惠民工程，举办第四届全民健身节、市民家庭趣味运动会，广泛开展全民健身运动；建设第十二届全运会比赛项目的相关设施，开放学校体育设施。四是加快发展老龄事业，努力实现老有所养、老有所乐、老有所为，让老年人共享改革发展成果。

　　更加注重社会管理。加快“数字抚顺”建设，强化应用系统开发，提高城市公共管理和公共服务的水平。创新社区管理机制，抓好社区自治、服务和组织体系建设。搞好“六五”普法宣传教育，健全社会治安防控体系，加强特殊人群管理，不断增强人民群众的安全感。畅通信访渠道，妥善解决合理合法诉求，及时提供法律援助，维护人民群众的合法权益。健全突发公共事件应急管理机制，提高应急反应和处置能力，加强防灾减灾体系建设。强化安全生产责任制，严格食品药品监管。加强诚信体系建设，打造诚信抚顺。

　　继续做好其它各项工作。

　　各位代表，民生就是人民的生存，民生就是人民的生活，民生就是人民的生命。关注民生是一种感情，倾注再多精力都不算多；保障民生是一种责任，做得多细、多实都不为过；改善民生是一种行动，给老百姓办的事都是对的，为老百姓花的钱都是值的。对各级政府而言，惠民富民的目标，没有最高，只有更高；对民生的投入，没有最多，只有更多；改善民生的追求，没有最好，只有更好。我们要尽最大努力，让社会更快发展、更加公正、更加和谐，让人民生活得更加殷实、更有尊严、更加幸福！

（九）努力建设人民满意政府

　　我们的政府是人民的政府，我们的权力来自人民，我们的天职是为人民服务。我们将勤勉尽责、永不懈怠，做到受人民监督、对人民负责、让人民满意。

　　进一步强化发展理念。当前，摆在全市面前的第一要务和中心任务，就是千方百计、全力以赴实现强劲发展，增强“等不起”、“慢不得”、“坐不住”的危机感、紧迫感和使命感，以战略思维和开放开阔的视野，协调好局部与整体、当前与长远、改革发展与和谐稳定的关系，推动经济社会持续快速发展。

　　进一步强化务实高效。大力弘扬奋勇争先的进取精神、迎难而上的拼搏精神、埋头苦干的务实精神。狠抓工作落实，对每一个项目、每一项工作都要明确责任、明确标准、明确进度，做到当日事当日毕。强化绩效考核，确保各项决策部署高标准推进、高质量完成。

　　进一步强化依法行政。自觉接受人大及其常委会的法律监督、工作监督和政协的民主监督，积极接受群众监督和舆论监督，主动听取民主党派、工商联、人民团体和社会各界人士的意见和建议，提高人代会议案、代表建议和政协提案的办理质量。不断提高政府的执行力和公信力，建设法治政府。

　　进一步强化廉政建设。高度警惕精神懈怠、能力不足、脱离群众和消极腐败的“四大危险”，加强党员干部廉洁从政教育，推进惩治和预防腐败体系建设。大力弘扬艰苦奋斗、勤俭办事的优良传统，把有限的资金用在发展和民生上。

　　各位代表，抚顺是我们的共同家园，建设和发展抚顺是我们的共同责任，任何困难都阻挡不了抚顺加快发展振兴的步伐，任何挑战都动摇不了我们建设幸福抚顺的决心！只要我们坚定信心，迎难而上，奋力拼搏，就一定能够迎来抚顺灿烂辉煌的明天！
